# Real-Time and Nanoplate-Based Digital PCR Assays for the Detection and Absolute Quantification of Borealpox Virus

**DOI:** 10.3390/ijms27031302

**Published:** 2026-01-28

**Authors:** Fabrizio Carletti, Francesca Colavita, Eliana Specchiarello, Valeria Ferraioli, Silvia Meschi, Enrico Girardi, Fabrizio Maggi

**Affiliations:** National Institute for Infectious Diseases Lazzaro Spallanzani I.R.C.C.S., 00149 Rome, Italy; fabrizio.carletti@inmi.it (F.C.); eliana.specchiarello@inmi.it (E.S.); valeria.ferraioli@inmi.it (V.F.); silvia.meschi@inmi.it (S.M.); enrico.girardi@inmi.it (E.G.); fabrizio.maggi@inmi.it (F.M.)

**Keywords:** Orthopoxvirus, Borealpox virus, molecular virology, digital PCR, real-time PCR, zoonosis

## Abstract

Borealpox virus (Alaskapox virus, BRPV), a neglected zoonotic Orthopoxvirus (OPXV), has been reported only in Alaska, usually causing mild infections. A recent fatal case in the Kenai Peninsula has raised concerns about its public health impact. Like other OPXVs, BRPV diagnosis relies on molecular tools, making species-specific assays essential. In this study, we developed and validated two BRPV-specific molecular assays: a TaqMan-based real-time PCR (rtPCR) and a digital PCR (dPCR) using the QIAcuity platform. Both target the viral CC-chemokines inhibitor (*vCCI*) gene, showing high sensitivity (limit of detection ~0.3–0.5 copies/μL), excellent specificity (no cross-reactivity with other OPXVs or rash-causing viruses), and strong reproducibility. While rtPCR is ideal for routine diagnostics, dPCR offers absolute quantification without standard curves, enhancing the detection of low viral loads. Although the lack of clinical BRPV-positive samples limits full validation, both assays show strong potential for improving BRPV detection, helping to distinguish it from other OPXVs and supporting the early identification of emerging orthopoxvirus threats.

## 1. Introduction

In recent years, members of the Orthopoxvirus (OPXV) genus have caused public health alarms due to an increase in OPXV infections worldwide. Recent outbreaks of monkeypox virus (MPXV), which led to a renewed public health emergency of international concern (PHEIC) declaration by World Health Organization (WHO) in 2024, have drawn attention to the threat of the emergence or re-emergence of OPXV species with potential human circulation [1]. The decline in immunity following the cessation of routine smallpox vaccination may be a factor associated with this phenomenon.

Among the recently identified Orthopoxviruses capable of infecting humans, the Borealpox virus (BRPV) represents one of the newest discoveries. This virus was originally named “Alaskapox”, after the first human case was identified in 2015 in a resident of Alaska. In 2024, following the first human fatality in the same U.S. state, the virus was renamed “Borealpox”, both to avoid geographical stigmatization and to reflect the mounting evidence that the virus has been circulating in populations of small mammals for decades, with its presence likely in boreal regions beyond Alaska. To date, seven BRPV cases have been reported to health officials, including one death of an immunocompromised patient [2]. A phylogenetic analysis of highly conserved genes revealed substantial divergence from known OPXV species, supporting the designation of BRPV as a distinct Orthopoxvirus species [3]. All cases had recent prior exposures to small mammals (shrews, voles, or squirrels), cats, and/or dogs. BRPV has been isolated in northern red-backed voles (Myodes rutilus). To date, the human-to-human transmission of BRPV has not been described [4].

The initial clinical manifestation of BRPV infection typically consists of pox-like cutaneous lesions. Cases present with a suspected spider or insect bite [4]. The physical examination usually reveals an ulcerated vesicle within an indurated erythematous patch. Other clinical manifestations, presenting a few days after the onset of the skin lesions, include fever, malaise, and lymphadenopathy. Most cases of BRPV infection have been mild, with a self-limited course of weeks to months [2].

Given that the clinical presentation overlaps with several other infectious and non-infectious syndromes, laboratory diagnosis is crucial to confirm BRPV disease. Molecular biology represents the first choice to detect OPXV infections [5,6,7]. Target-specific approaches and multiplex assays based on conserved viral genes have been developed [6,7]. Following the global mpox outbreak, commercial assays are now available for MPXV detection, while in-house methods are used for the other OPXV species.

Here, we report the set-up of a novel real-time PCR (rtPCR) assay and its optimization to the nanoplate-based digital PCR system able to detect and quantify BRPV DNA. We describe the assessment of the analytical performance of the novel assays using BRPV isolate and investigate the specificity against other related and non-related OPXV species.

## 2. Results

### 2.1. Analytical Sensitivity

**Figure 1:**
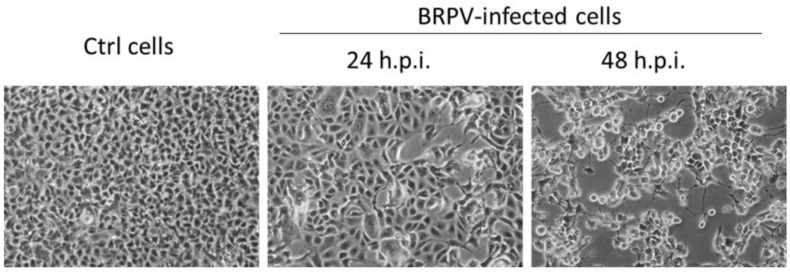
BRPV culture in Vero E6 cells. Mock-infected (**left**) and infected Vero E6 cells with BRPV strain (**right**) observed after 24 and 48 h post-infection (h.p.i.). Bright-field images acquired with a 20× objective. Scale bar: 100 µm.

The analytical sensitivity of the BRPV rtPCR and dPCR assays was evaluated using the BRPV DNA extracted from culture supernatant (Figure 1). The titer of the viral stock used was Log_10_ 6.42 TCID50/mL.

The viral DNA was tested in serial dilutions to achieve 12 serial dilutions from 5.42 to 0.42 Log_10_TCID50/mL. As shown in Table 1, 6 to 15 replicates were performed. A Ct > 40 was considered negative for the rtPCR assay, while dPCR was considered negative when a signal was detected from fewer than three partitions.

**Figure 2:**
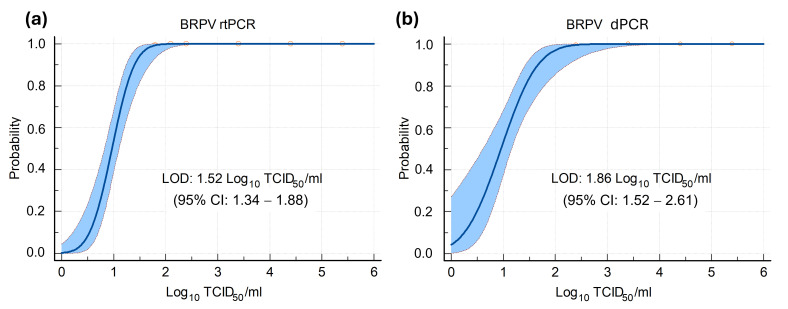
Probit analysis for BRPV (**a**) rtPCR and (**b**) dPCR. Limit of detection (LOD) and 95% CI are expressed as Log_10_ of 50% tissue culture infectious dose (TCID50)/mL. The LOD was calculated using the replicate numbers reported in Table 1 (for each dilution).

Probit regression analysis was used to determine the limit of detection (LOD) of the assays at 95% confidence, defined as the lowest viral concentration measured with a 95% hit rate in replicates of serial dilutions of viral DNA extracted from the culture supernatant. As shown in Figure 2, the LOD was 1.52 (95% CI: 1.34–1.88) and 1.86 (95% CI: 1.52–2.61) Log_10_TCID50/mL for rtPCR and dPCR, respectively. According to the DNA quantitation by dPCR, the LOD TCID50/mL values correspond to about 0.3 and 0.5 DNA copies/µL, respectively.

Of note, BRPV DNA levels quantified by dPCR (copies/µL) showed a strong inverse correlation with the Ct values obtained from the rtPCR assay (r = −0.9939, 95% CI: −0.9996 to −0.9074; *p* = 0.0006).

### 2.2. Assessment of Intra- and Inter-Run Variability

Both intra-run (repeatability) and inter-run (reproducibility) variabilities of the rtPCR were assessed using two dilutions of the BRPV isolate—1:100 and 1:100,000. Intra-run variability was assessed by performing nine repeated measurements on the two dilutions, each containing 315.7 copies/μL and 0.5 copies/μL, respectively. Inter-run variability was evaluated by analyzing two different dilutions in two independent runs. The percentage coefficient of variation (CV) for intra-run variabilities ranged from 0.27% (range Ct 23.66 to 24.25) to 1.49% (range Ct 33.08 to 35.02). The percentage coefficient of variation (CV) for inter-run variabilities was assessed to be 0.82%.

### 2.3. Specificity Evaluation

**Figure 3:**
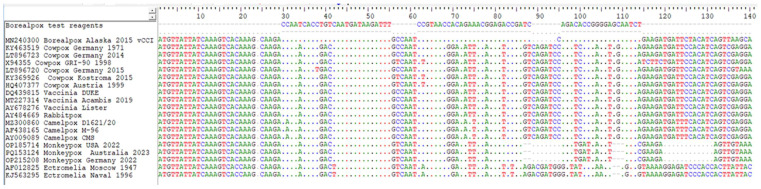
Alignment of primers and probes with OPXV sequences. NCBI accession numbers are shown for each OPXV sequences included in the analysis. Different colours are used to identify the nucleotide sequence alignment (Thymine: red; Adenine: green; Guanine: black; Cytosine: blue).

The analytical specificity of the rtPCR assay was first determined by sequence alignment between BRPV strains and other Orthopoxviruses (Figure 3). This analysis showed that the selection of the viral CC-chemokines inhibitor (*vCCI*) gene as molecular target allowed the assay to only detect BPRV, without cross-reactivity with other viruses.

The analytical specificity was then evaluated against other Orthopoxviruses (Table 2), using viral stock preparations (*n* = 4), clinical residual samples (*n* = 1) and residual viral extracted DNA (*n* = 4). The assay did not react with the nine different Orthopoxviruses included in the test panel, showing a specificity of 100%. In addition, three clinical samples collected from patients positive for other rash illnesses (i.e., HSV, VZV) and one negative from a healthy donor were tested for clinical specificity. The BRPV rtPCR assay was confirmed to be 100% specific, based on the absence of non-specific signal detection due to any type of cross-reactivity event.

### 2.4. Signal Intensity

**Figure 4:**
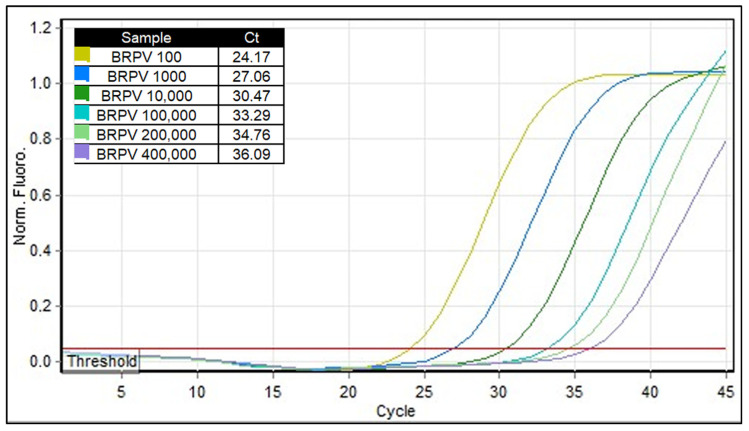
Intensity of fluorescence signal of high and low BRPV DNA copies. Amplification curves on Rotor Gene Q (Qiagen, Hilde, Germany) platform of 6 different BRPV DNA dilutions (1:100, 1:1000, 1:10,000, 100,000, 1:200,000, 1:400,000). The corresponding Ct values are shown in the inset table.

Signal intensity is an important parameter for rt-PCR methods, as it ensures a clear distinction between positive and negative results. Weak signals can occur when testing samples containing low levels of genome copies. As shown in Figure 4, the BRPV rtPCR also provided a strong and clear signal of amplification for those samples with Ct values close to the limit of detection.

## 3. Discussion

The OPXV genus includes a variety of viruses, some of which affect humans and various wild and domestic mammals. The most important of these are vaccinia (VACV), cowpox (CPXV), camelpox (CMPV), horsepox (HPXV), monkeypox (MPXV) and BRPV [8]. Following the eradication of smallpox and the subsequent cessation of smallpox vaccination, there has been a steady increase in OPXV infections causing zoonoses in humans [5]. As shown by the global spreading of MPXV starting in 2022, concerns about the potential for OPXV outbreaks with serious public health implications warrant close monitoring and preparedness efforts. Among the OPXVs, BRPV infections are so far confined to the Alaskan region, with sporadic human cases reported and mild disease in immunocompetent people [2,4]. However, the recent news about the death of a man living in the Kenai Peninsula, AK, USA drew attention to this neglected zoonotic OPXV, underlining the need for increased surveillance and research [9]. The genomic sequencing of the BPRV isolated in 2015 revealed that the virus more closely resembles Old World Orthopoxviruses (92.9% sequence similarity) than New World OPVs (87.1%), with the closest relative being the Akhmeta virus (AKMV), which was isolated in 2013 in Georgia (93.5%) [10].

The confirmation of OPXV infections relies on the detection of viral DNA [5]. Diagnostic tests capable of distinguishing between different Orthopoxvirus species are particularly valuable, as they significantly enhance the accuracy and specificity of the diagnosis [11,12]. While pan-Orthopoxvirus assays are effective at detecting OPXV DNA, they lack species-level resolution; therefore, additional genomic characterization is required to accurately identify the species [12]. The global spread of MPXV with novel viral sub-clades that hinder detection capabilities has highlighted the urgent need for new diagnostic tools capable of distinguishing between different OPXVs [13,14].

Here, we report the in-house protocols for the specific detection of BRPV DNA using both TaqMan-based rtPCR and dPCR approaches. The latter represents a new generation of PCR technology that has gained increasing attention in the field of molecular virology for its application in virus detection. Compared to the widely used rtPCR, dPCR enables the absolute determination of nucleic acid copies without the use of standard curves, providing the exact concentration of the target genomic material in biological samples. As demonstrated in our previous work on nanoplate-based digital PCR assay for MPXV [15], the QIAcuity dPCR platform enables the adaptation of gene amplification protocols, providing a robust, precise, and user-friendly method for quantifying viral DNA across diverse biological samples. Both assays demonstrated robust analytical sensitivity for the detection of BRPV DNA, and no cross-reactivity was observed against other viruses, including other related OPXV and rash illness viruses such as HSV-1, HSV-2 and VZV involved in the differential diagnosis. In detail, both methods were able to detect genomes down to 1 copy/μL (approximately 0.3 and 0.5 DNA copies/μL for rtPCR and dPCR, respectively). Extensive analysis was performed using rtPCR as the reference method. No false-positive results were observed during the evaluation of either viral isolates or clinical specimens, resulting in 100% specificity. In addition, the repeatability and reproducibility of the assay were robust across different viral concentrations. Intra-run variability, assessed through nine replicates of two dilutions, exhibited a low CV (ranging from 0.27% to 1.49%), while inter-run variability also remained within acceptable limits (CV = 0.82%), indicating consistent assay performance across different runs and days. These values highlight the high precision and technical reliability of the rtPCR assay, even when detecting low copy numbers near the LOD.

Overall, targeting the *vCCI* gene enabled selective and highly specific detection, with both methods demonstrating high sensitivity and excellent reproducibility. The results support their potential use for BRPV genome detection in both research and diagnostic settings. These assays expand the available tools for diagnosing and monitoring of neglected OPXV infections, including BRPV. In addition, the adaptation of the test to the dPCR approach allows the absolute quantification of nucleic acid copies with the ability to detect low genomic levels.

Of note, the availability of a single BRPV sequence and the lack of real clinical samples from BRPV-infected patients limit our study and require the further validation of our assays to support their application in clinical and epidemiological settings. In addition, it should be noted that targeting the genomic termini may render the assays susceptible to mutations; therefore, the new assays may be informative for viral characterization or as reflex tests when used in combination with other Orthopoxvirus assays. In conclusion, the possibility of OPXV human-to-human transmission is a serious concern for the national and global health organizations. Although many of these viruses, such as BRPVs, are currently confined to limited geographic areas, the continued emergence and potential for expansion of OPXV species underscore the critical need for continuous surveillance and significant improvements in diagnostic methods. Enhancing diagnostics is essential for early detection and timely interventions to control transmission.

## 4. Materials and Methods

### 4.1. Biological Samples

The BRPV isolate (accession number: MN240300; [3]), kindly provided by the United States’ Centers for Diseases Control and Prevention (U.S. CDC, Atlanta, GA, USA), was used to assess the analytical sensitivity of the novel assays. Virus was grown in Vero E6 cells maintained in Minimum essential Medium (Corning, NY, USA) supplemented with 1% Penicillin/1% Glutammin and 10% Fetal Bovine Serum, at 37 °C in a 5% CO_2_ atmosphere. For the stock preparation, Vero E6 cells were exposed to the BRPV strain for 1 h at 37 °C, at a multiplicity of infection (MOI) of 0.1. Cytopathic effect (CPE) appearance was observed by a light microscope from 24 h post-infection (Figure 1). Virus stock titer was obtained by limiting dilution assay on the Vero E6 cell line and expressed as 50% tissue culture infectious dose Log_10_TCID50/mL, according to the Reed and Muench method [16].

The panel of samples used for the specificity analysis included viral stocks and residual clinical samples (Table 2). All viral strains were prepared as described above and tested diluted in molecular grade water; residual clinical samples were stored at the Laboratory of Virology of the National Institute for Infectious Diseases (INMI) “Lazzaro Spallanzani” I.R.C.C.S. (Rome, Italy) following the completion of the diagnostic routine activities.

### 4.2. DNA Extraction

Viral DNA from viral stocks and clinical samples was isolated using the QIAmp Viral DNA Mini kit (Qiagen, Hilden, Germany), following the manufacturer’s instructions. The extracted nucleic acids were eluted in 200 µL of elution buffer (Qiagen, Germany) and stored at −80 °C until use.

### 4.3. Real-Time PCR Assay Protocol

The rtPCR assay was designed to target the viral CC-chemokines inhibitor (*vCCI*) gene of BRPV. This gene was chosen as the molecular target because it includes a BRPV sequence signature. The primers and probe were designed within a region that is divergent from other OPXVs, as illustrated by the comparative alignment in Figure 3. A multiple-sequence alignment was performed between the BRPV sequence and representative sequences of the other Orthopoxviruses. The design was carried out using the AlleleID software v 7.85 (Premier Biosoft, San Francisco, CA, USA). The sequences of the primers and probe of the assay are reported in Table 3. A 10 µL volume of extracted DNA, or RNAse-free water as negative control, was added to 20 µL of mix containing the following reagents: 4.1 µL of nuclease-free water, 15 µL of 2× GoTaq Probe qPCR mastermix (Promega corporation, Medison, WI, USA), 0.6 µL of 25 µM primer mix (final concentration: 0.5 µM) and 0.3 µL of 20 µM of Taqman probe (final concentration: 0.2 µM). The amplification reaction was performed on a Rotor Gene Q (Qiagen, Hilden, Germany) using the following cycling conditions: 95 °C for 2 min, followed by 45 cycles of 95 °C for 15 s and 58 °C for 60 s. Amplification curves were evaluated with a threshold placed above overt background signal; results of cycle threshold (Ct) values > 40 were considered negative.

### 4.4. dPCR Assay Protocol

The dPCR reaction was performed using the QIAcuity One Platform System 5-plex and the QIAcuity Nanoplate (Qiagen, Hilden, Germany), 26k 24-well, allowing an average of 25,000 reactions for each sample. The mixture for BRPV was prepared using primers and probe concentrations according to the manufacturer’s instructions: 10 μL of 4× UCP Probe PCR Kit, 1 µL of 25 µM primer mix (final concentration: 0.5 µM) and 0.3 µL of 20 µM of Taqman probe (final concentration: 0.2 µM). For the BRPV *vCCI* gene, 10 μL of DNA and RNase-free water (as a negative control) were mixed to reach a final reaction volume of 40 μL.

The PCR mix was prepared in a pre-plate and then transferred into the 24-well 26 K nanoplate. The QIAcuity One is a fully automated platform system whose workflow includes the priming and rolling step in order to generate and seal the single partitions; the amplification step: 95 °C for 2 min, 95 °C for 15 s and 58 °C for 40 s for 40 cycles; and the final imaging step, done by reading BRPV through the FAM channel. Data were analyzed using the QIAcuity Suite Software V1.1.3 193 and the DNA amount was expressed as copies/μL.

### 4.5. Statistics

The limit of detection (LOD) at the 95% confidence interval (95% CI) was calculated using probit regression analysis with MedCalc statistical software (version 9). The correlation analysis between Ct values obtained by the rtPCR and copies/µL measured by the dPCR assay were performed using GraphPad Prism V 9 (San Diego, CA, USA) statistical package.

## Figures and Tables

**Table 1:** Analytical sensitivity of rtPCR and dPCR assays across dilutions of BRPV viral particles.

Viral Stock Dilution (Log_10_TCID50/mL)	rtPCR			dPCR		
	%Detection (reps)	Mean Ct	SD	%Detection (reps)	Mean DNA cp/μL	SD
1:10 (5.42)	100% (6/6)	20.9	0.3	100% (6/6)	2814.6	75.8
1:100 (4.42)	100% (9/9)	24.0	0.2	100% (9/9)	315.7	35.6
1:1000 (3.42)	100% (9/9)	27.4	0.4	100% (9/9)	29.5	6.0
1:10,000 (2.42)	100% (9/9)	30.6	0.4	100% (9/9)	3.4	6.0
1:20,000 (2.12)	100% (6/6)	32.1	0.5	100% (6/6)	1.3	0.3
1:40,000 (1.82)	100% (6/6)	32.7	0.3	100% (6/6)	0.5	0.2
1:80,000 (1.52)	100% (6/6)	34.1	0.9	83.3% (5/6)	0.3	0.2
1:100,000 (1.42)	86.7% (13/15)	33.9	0.7	73.3% (11/15)	0.5	0.4
1:200,000 (1.12)	50% (6/12)	34.8	0.7	50% (6/12)	0.4	0.1
1:400,000 (0.82)	58.3% (7/12)	35.1	0.5	55.5% (5/9)	0.3	0.1
1:800,000 (0.52)	0% (0/9)	-	-	33.3% (1/3)	0.4	-
1:1,000,000 (0.42)	0% (0/9)	-	-	0% (0/3)	-	-

Abbreviation: Ct, cycle threshold; reps, replicates; SD, standard deviation; rtPCR, real-time PCR; dPCR, digital PCR; TCID50/mL, 50% tissue culture infectious dose per milliliter.

**Table 2:** Biological samples tested to assess specificity of the BRPV rtPCR assay.

Type of Specimen		Virus/Strain	Input Concentration ^§^	BRPV rtPCR Assay
Viral stock		MPXV clade 1a	Ct: 29	Not detected
		MPXV clade 1b	Ct: 27	Not detected
		MPXV clade 2b	Ct: 27	Not detected
		Camelpox virus	Ct: 28	Not detected
Residual QC DNA *		Cowpox virus BR	Ct: 31	Not detected
		Ectromelia	Ct: 27	Not detected
		Vaccinia virus WR	Ct: 27	Not detected
		Vaccina virus MVA	Ct: 30	Not detected
Clinical sample **	Serum	HSV1	1476 cp/mL	Not detected
	Skin lesion	HSV2	77,322 cp/mL	Not detected
	Liquor	VZV	141,633 cp/mL	Not detected
	Skin lesion	Cowpox/Feline poxvirus	Ct: 26	Not detected
	Blood, plasma	Negative (healthy donor)	-	Not detected

* Extracted nucleic acid leftovers from international EQA initiatives in which INMI participated (2019); samples were stored in liquid nitrogen. ** Clinical samples left over from the diagnostic routine activities at INMI; samples were stored at −80 °C and anonymized prior to their use. **^§^** Data refers to the diagnostic methods specific to the tested virus/strain.

**Table 3:** Primers and probe used in the BRPV assays.

Primer/Probe	5′ --> 3′ Sequence	Target	Nt Position ^a^ (Minus Strand)	Amplicon Size
Forward	CCAATCACCTGTCAATGATAAGATTT	*vCCI*	141–166	78 Nt
Reverse	AGATTGCTCCCCGGTGTCT		219–201	
Probe	FAM-CCGTAACCACAGAAACGGAGACCGATC-BHQ		173–199	

^a^ Refers to the sequence of *vCCI* gene of MN240300. Abbreviations: Nt, nucleotide; *vCCI*, viral CC-chemokine inhibitor.

## Data Availability

The data supporting the findings are available from the corresponding author upon reasonable request, but only for sections that do not contain infringing personal information.
